# Folding and Oligomerization of the gp2b/gp3/gp4 Spike Proteins of Equine Arteritis Virus *in Vitro*

**DOI:** 10.3390/v4030414

**Published:** 2012-03-22

**Authors:** Aleksander Kabatek, Michael Veit

**Affiliations:** Department of Immunology and Molecular Biology, Veterinary Faculty, Free University, Philippstraße 13, Berlin 10115, Germany; Email: ajk21@gmx.de

**Keywords:** equine arteritis virus, glycoproteins, gp2b, gp3, gp4, protein folding, oligomerization

## Abstract

Equine arteritis virus (EAV) is a small, positive-stranded RNA virus. The glycoproteins gp2b, gp3 and gp4 form a heterotrimer in the viral envelope, which is required for cell entry of EAV. We describe expression of the ectodomains of the proteins in *E. coli* and their refolding from inclusion bodies. After extraction of inclusion bodies and dialysis, Gst-, but not His-tagged proteins, refold into a soluble conformation. However, when dialyzed together with Gst-gp3 or with Gst-gp4, His-gp2b and His-gp4 remain soluble and oligomers are obtained by affinity-chromatography. Thus, folding and oligomerization of gp2b, gp3 and gp4 *in vitro* are interdependent processes.

## 1. Introduction

Equine arteritis virus (EAV) is a positive-stranded RNA virus belonging to the family *arteriviridae* of the order *nidovirales* [1]. EAV enters cells by clathrin-mediated endocytosis [2] and newly formed virus particles bud into intracellular membranous compartments of the exocytic pathway. EAV contains seven structural proteins: the nucleocapsid protein N and six membrane proteins, the glycoproteins gp2b, gp3, gp4, gp5 and the unglycosylated proteins E and M [3]. The membrane proteins form disulfide-linked heterodimeric (gp5/M) or heterotrimeric complexes (gp2b/gp3/gp4), but E is present as monomer [4,5,6]. N, M, and gp5 are major virion components, E occurs in virus particles in intermediate amounts, and gp2b, gp3 and gp4 are minor structural proteins. 

All of the structural proteins are essential for virus infectivity, but their precise function during the replicative cycle of EAV is not known. Gp5, M and N, but not E, gp2b, gp3, or gp4, are required for budding of virions, implicating the latter in virus entry [7]. Likewise, exchanging the ectodomains of gp5 and M of EAV with those of other arteriviruses does not change the cell tropism of the recombinant virus [8,9]. Thus, the recently described gp2b/gp3/gp4 complex is currently a prime candidate for the receptor-binding and the membrane-fusion activity of EAV. For this purpose the gp2b/gp3/gp4 complex probably interacts structurally or at least functionally with the myristoylated E‑protein [5,10]. Removal of E from the EAV genome completely prevents incorporation of the gp2b/gp3/gp4 complex into virus particles, whereas in the absence of the gp2b/gp3/gp4 complex the amount of E present in virus particles is greatly reduced [7].

Gp2b and gp4 are typical type I membrane proteins with a cleavable N-terminal signal peptide and a C-terminal hydrophobic transmembrane domain. In contrast, cleavage of the signal peptide from gp3 was not observed and it is thus possible that the protein is membrane-anchored by both of its hydrophobic terminal domains [7,11]. In the lumen of the endoplasmic reticulum (ER), gp2b, gp3 and gp4 are N-glycosylated and a disulfide-linkage is formed between gp2b and gp4. Gp2b, gp3 and gp4, either expressed individually or from the viral genomem, are retained in the ER. After release of virus particles from the infected cell a further and unique protein modification occurs. The gp2b/gp4 dimer is rapidly and spontaneously converted into a disulfide-bonded gp2b/gp3/gp4 trimer [12]. Since formation of a disulfide-linkage requires the close proximity of the two participating cysteines of gp3 and gp4, it is likely that a non-covalently linked gp2b/gp3/gp4 trimer exists prior to disulfide-bond formation, but this has not been verified experimentally.

Oligomerization and folding of viral transmembrane proteins are usually interdependent processes. Proper folding of a protein is often dependent on the presence of a binding partner from the oligomer, but on the other side oligomerization requires the recognition of structural motifs present only in (at least partially) folded protein domains. Folding starts cotranslationally when the first prospective domain is completely translocated into the lumen of the ER and is aided by the addition of N-linked carbohydrates and by the formation and subsequent remodeling of disulfide-linkages. A quality control system finally ensures that misfolded proteins are retained in the ER and eventually degraded to prevent their exposure at the cell surface [13,14,15]. Since many cellular proteins and enzymes, such as oligosaccharide transferases, disulfidisomerases and the elements of the quality control system are required for folding and oligomerization of proteins in the lumen of the ER, this process is often difficult to reconstitute with recombinant and purified proteins, especially when they were purified from *E.coli*, which lack most enzymes for protein modification. Recently we have expressed Gp2b, gp3 and gp4 with the baculovirus system in insect cells, but the expression levels were very low, precluding further functional and structural studies with purified proteins [16]. Here we have expressed the ectodomains of gp2b, gp3 and gp4 in *E. coli*. and analyzed folding and oligomerization of a gp2b/gp3/gp4 complex. 

## 2. Results

### 2.1. The Ectodomains of Gp2b, Gp3 and Gp4 Are Insoluble When Expressed in E. coli

**Figure 1:**
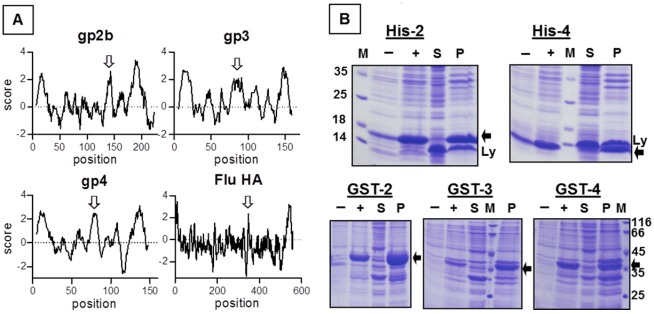
Expression of the ectodomains of gp2b, gp3 and gp4 in *E. coli* and their accumulation in inclusion bodies. (**A**) Kyte-Doolittle hydropathy plots (window size 9) for gp2b, gp3, gp4 and for the hemagglutinin of influenza virus (Flu).The hydrophobicity score is plotted against the amino acid residue (position) of the respective protein. Scores above 2 are considered to be highly hydrophobic, *i.e.*, are likely to insert into membranes. The N- and C-terminal hydrophobic regions are the proposed signal peptides and the transmembrane regions, respectively. The third hydrophobic region in the middle of the molecule, which has been shown to function as fusion peptide in the case of HA, is marked with a white arrow. The constructs used in this study contain the complete ectodomain of gp2b, gp3 and gp4, *i.e.*, excluding the N- and C-terminal hydrophobic region. (**B**) Expression of 6 × His-tagged (upper panel) and Gst-EAV-proteins (lower panel) in *E.coli* and their accumulation in inclusion bodies. *E. coli* containing the respective expression plasmids were induced with IPTG and were grown overnight at room temperature. Pelleted cells were resuspended in lysis-buffer, lysed by sonication and centrifuged for 20 min at 5,000 × g. Aliquots of cell lysates before (–) and after (+) induction of protein synthesis as well as supernatants (S) and pellets (P) were subjected to SDS‑PAGE under reducing conditions and Coomassie staining. The expressed EAV‑proteins are marked with a black arrow. M: molecular mass markers as indicated, Ly: Lysozyme (14 kDa), which is added before preparation of cell lysates.

To express the EAV-glycoproteins in *E. coli* in a form likely to be soluble, it might be necessary to delete hydrophobic domains. Hence, we performed Kyte-Doolittle plots with gp2b, gp3 and gp4 to identify their hydrophobic regions [17]. Surprisingly, besides the described N- and C-terminal hydrophobic domains, which are likely to function as signal peptide or transmembrane region, all three glycoproteins contain another hydrophobic region in the middle of the molecule (Figure 1A). This pattern of hydrophobic domains is reminiscent of viral fusion proteins, such as the hemagglutinin of influenza virus, where the third hydrophobic domain functions as a fusion peptide, which is exposed at the surface of the molecule and inserts into the cellular membrane catalysing its fusion with the viral membrane [18,19]. The occurrence of three hydrophobic domains in each EAV-glycoprotein suggests that each contains a fusion peptide and thus supports the concept that the gp2b/gp3/gp4-complex is involved in membrane fusion.

We cloned DNA sequences encoding the ectodomains of gp2b, gp3 and gp4 into pQE expression-plasmids. The resulting constructs contained a 6xHis affinity tag instead of the N-terminal signal peptide and lacked the C-terminal transmembrane region. Expression of His-gp2b and His-gp4 in *E. coli* was clearly detectable by SDS-PAGE and Coomassie-staining of cellular extracts (Figure 1B), but His-gp3 was not expressed at all (data not shown). When cellular extracts were separated by centrifugation into a soluble and a particulate fraction prior to SDS-PAGE, it became obvious that both His-gp2b and His-gp4 are completely insoluble. We therefore replaced the N-terminal histidines by glutathione-S-transferase (Gst), which is supposed to increase the solubility of proteins to which it is attached. Nevertheless, Gst-gp2b and Gst-gp4 were still exclusively present in the particulate fraction of *E. coli*. However, fusion to Gst instead to 6xHis now allows expression of gp3, but Gst-gp3 was also completely insoluble (Figure 1B). Several attempts to enhance the solubility of the EAV-proteins, *i.e.*, by reducing the expression time or temperature or the amount of IPTG used for induction, were not successful (not shown). Thus, all three glycoproteins do not fold into a soluble conformation when expressed in *E. coli*, but instead are deposited as insoluble aggregates in inclusion bodies. However, recent data suggested that inclusion bodies do not necessarily contain “useless”, *i.e.*, misfolded proteins, but proteins present in alternative conformational states [20,21].

### 2.2. Refolding of the Ectodomains of Gp2b, Gp3 and Gp4 from Inclusion Bodies

**Figure 2:**
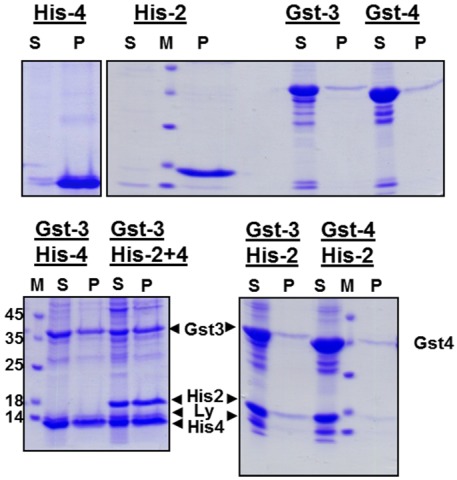
Refolding of EAV proteins during dialysis of urea-extracts of inclusion bodies. Upper panel: Dialysis of individual EAV-proteins. Lower panel: Combined dialysis of the indicated combinations of EAV-proteins. Inclusion bodies were resuspended in denaturating buffer. After centrifugation the resulting supernatants were dialyzed against buffer with decreasing urea concentration and finally against physiological buffer. The dialysate was then centrifuged and aliquots of the supernatant (S) and of the pellet (P) were analyzed by SDS-PAGE under reducing conditions and Coomassie-staining. M: molecular mass marker as indicated, Ly: Lysozyme. Due to the lack of antibodies we were not able to confirm the identiy of the bands by Western blot. Nevertheless, since the recombinant proteins are the major proteins present in bacterial lysates (see Figure 1), we are sure that the identification of bands is correct.

The inclusion bodies were extracted with denaturating agents (2 M urea, pH 12.5) to dissolve the aggregates and unfold the proteins. To (possibly) allow refolding of proteins [22], incompletely solubilized components were pelleted and the resulting supernatant was subjected to stepwise dialysis against buffers containing decreasing urea concentration and finally against physiological phosphate-buffer. To analyze whether the solubilized protein were precipitated or remained soluble during this procedure, each dialysate was centrifuged and the resulting supernatant and pellet were subjected to SDS-PAGE. Whereas His-gp2b and His-gp4 completely precipitated during dialysis, Gst-gp3 and Gst‑gp4 remained soluble indicating that the Gst-tag facilitated folding of the proteins into a soluble conformation (Figure 2, upper panel). In contrast, refolding of GST-Gp2b was very inefficient (not shown).

Transmembrane proteins forming a heterooligomeric spike in virus particles often require each other for proper folding inside cells [14,15]. To analyze whether folding of His-gp2b and/or His-gp4 might be positively affected by the presence of Gst-gp3 or Gst-gp4 *in vitro*, we combined various supernatants obtained from the urea extracts of inclusion bodies prior to dialysis. Strikingly, His-gp2b as well as His-gp4 remain (for most parts or even completely) soluble when dialyzed together with Gst-gp3 or with Gst-gp4 (Figure 2, lower panel). Thus, His-gp4 as well as His-gp2b can fold into a soluble conformation when either Gst-gp3 or Gst-gp4 is present during dialysis.

### 2.3. Oligomerization Is Required for Refolding of the Ectodomains of Gp2b, Gp3 and Gp4

**Figure 3:**
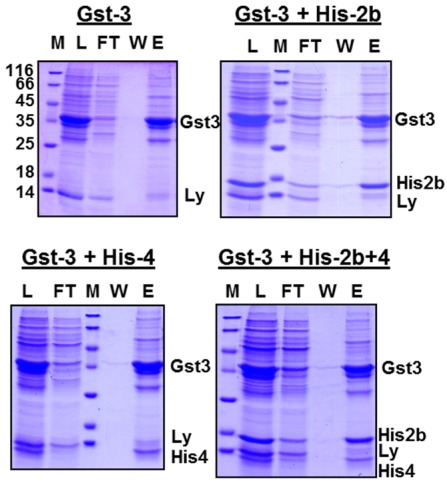
Affinity-purification of EAV-protein oligomers. Gst-gp3, His-gp2b and His-gp4 were expressed individually in *E. coli* and solubilized from inclusion bodies, and refolded by dialysis. Urea extracts from inclusion bodies containing Gst-gp3 were dialysed alone (Gst-3) or were combined with His-gp2b (Gst-3+His-2b), with His-gp4 (Gst-3His-4) or with both (Gst-3+His-2b+4) prior to dialysis. The cleared dialysate was then agitated with glutathione-beads, beads were pelleted, put in a column, and washed 5 times with phosphate-buffered saline. Proteins were eluted with elution buffer. Aliquots of the dialysate (load), of the flow-through (FT), of wash 5 (W5) and of the eluates (E) were subjected to SDS-PAGE under reducing conditions and Coomassie-staining. M: Molecular mass markers, Ly: Lysozyme.

We tested whether oligomerization of EAV-proteins occurs during refolding. After dialysis of Gst‑gp3, either alone or in combination with His-gp2b, or with His-gp4, or with both proteins, the cleared dialysate was loaded onto a glutathione-column, the column was washed, and bound proteins were eluted with glutathione. SDS-PAGE of these fractions shows that the His-tagged proteins are present together with Gst-gp3 in the eluate for all combinations (Figure 3). This must be due to the formation of oligomers during refolding, since lysozyme, which is added before lysis of bacteria, is present in the fraction which was loaded onto the column, but largely removed during affinity chromatography. However, it cannot be inferred from the data of the experiments involving all three EAV-proteins, whether a heterotrimeric complex or two heterodimers, *i.e.*, Gst-gp3/His-gp2b and Gst‑gp3/His-gp4, have been formed. Since the complete trimeric Gp2b/3/4 complex is very unstable, e.g., it does not resist purification by centrifugation through a sucrose gradient [12], we were not able to clarify this important question.

## 3. Experimental Section

### 3.1. Expression of the Gp2b, Gp3 and Gp4 Ectodomains of EAV in *E. coli*

Nucleotides encoding the ectodomains of Gp2b (amino acids 25 to 169), Gp3 (amino acids 24 to 138) and Gp4 (amino acids 22 to 125) from the Bucyrus strain of equine arteritis virus were amplified with PCR from pBluescript KS plasmids and subcloned into pQE30 and pGEX-6P-1. As a result, each protein contains either a 6xHis or a GST-tag at its N-terminus. 

*E. coli* cells (strain BL21 and XL1-blue) containing the respective expression plasmids (pQE-30 and pGEX-6P-1) were induced at an OD_600_ of 0.8 with 0,1 mM IPTG and were grown overnight at room temperature. Pelleted cells (10 min, 10.000 × g) were resuspended in lysis-buffer (20 mM Tris, (pH 7.4), 150mM NaCl), lysed on ice by sonication with a pobe-type sonicator and lysozyme (final concentration of 1 mg/mL) and DNAse I (final concentration of 40 µg/mL)) were added. The samples were incubated on ice for 30 minutes and centrifuged for 20 min at 5,000 × g. Aliquots of cell lysates before and after induction of protein synthesis as well as supernatants (containing soluble proteins) and pellets (inclusion bodies containing insoluble proteins) were subjected to SDS-PAGE (reducing conditions, 15% polyacrylamide gels) and Coomassie staining. 

### 3.2. Refolding of the Gp2b, Gp3 and Gp4 Ectodomains from Inclusion Bodies

Inclusion bodies, prepared as described above, were resuspended in denaturating buffer (100 mM Tris, (pH 12.5), 2 M urea, 10 mL buffer was used for cells pelleted from 100 mL medium) and agitated for 1 hour at 4 °C. After centrifugation (10 min, 40,000 × g) the resulting supernatants were dialyzed (Slide-A-Lyzer with 10 kDa molecular weight cut-off), firstly for one hour against 50 mM Tris (pH 10.5), 1M urea, 5% glycerol, 2.5% sucrose, secondly for one hour against 50 mM Tris (pH 9.0), 0,5M urea, 7.5% glycerol, 3.75% sucrose), and finally overnight against phosphate-buffered saline (pH 8) at 4 °C. The dialysate was then centrifuged (1 min, 20,000 × g) and aliquots of the supernatant (containing soluble proteins) and of the pellet (containing insoluble proteins) were analyzed by SDS‑PAGE under reducing conditions and Coomassie-staining.

### 3.3. Purification of the Gp2b, Gp3 and Gp4 Oligomers

Gst-gp3, His-gp2b and His-gp4 were expressed individually in *E. coli* and solubilized from inclusion bodies as described above. Urea extracts from inclusion bodies containing Gst-gp3 were dialysed alone or were combined with His-gp2b, with His-gp4 or with both His-gp2b and His-gp4 prior to dialysis. The cleared dialysate was agitated with glutathione-beads for one hour, beads were then pelleted (1 min, 1,000 × g), put in a column, and washed 5 times with phosphate-buffered saline (pH 8). Proteins were eluted with elution buffer (10 mM reduced glutathione in phosphate-buffered saline, pH 8). Aliquots of the dialysate, of the flow-through, of wash 5 and of the eluates were subjected to SDS-PAGE under reducing conditions and Coomassie-staining.

## 4. Discussions and Conclusions

In summary, we have shown that folding of the recombinant ectodomains of gp2b and gp4 *in-vitro* is enhanced by the presence of another component of the heterotrimeric complex and that the beneficial effect is due to the formation of oligomers. Thus, oligomerization and folding are interdependent processes, being in line with current knowledge on the formation of spike proteins of enveloped viruses, which, however, were mainly studied inside cells [14,15]. 

The gp2b/gp3/gp4 complex is one of the few described virus spikes composed of three different proteins. Thus, pathways leading to trimerization, *i.e.*, whether it occurs via stable dimeric intermediates have not been studied so far. We have observed the formation of all possible combination of dimers, e.g., gp2b with gp3 and with gp4 as well as gp3 with gp4. It is thus likely that the first step in oligomerization is formation of a dimer, which then recruits the third protein to build a trimer. Since gp2b, gp3 and gp4 are abundantly expressed in the cell, but only sparsely incorporated into virus particles, it is likely that not each combination of dimers leads to the formation of a functional trimeric spike. 

Surprisingly, disulfide-bonds, either intramolecular in gp2b or intermolecular between subunits of the gp2b/gp3/gp4-complex, which have an important function for incorporation of the gp2b/gp3/gp4 complex into virus particles and especially for virus infectivity [6,11,12], are not essential for folding and oligomerization of the gp2b/gp3/gp4 complex, at least *in vitro*. However, since the formation of disulfide-bonds requires close proximity of the cysteines involved, proper folding might be a prerequisite for disulfide-bond formation. Likewise, N-linked carbohydrates are not required for folding and oligomerization, but the Gst-tag can apparently replace the glycosylation in its function of increasing the solubility of folding intermediates. Thus, glycosylation has no structural role, but *in vivo* it is most likely required for interaction of gp2b, gp3 and/or gp4 with elements of the quality control system [13,15]. Likewise, the transmembrane region (which was deleted from each construct in our study), especially if embedded in its natural lipid environment, might affect folding and oligomerization of the gp2b/gp3/gp4-complex. It thus remains to be shown whether the described folding and oligomerization pathway applies also to formation of the gp2b/gp3/gp4 complex in virus‑infected cells. 

## References

[1] MacLachlan N.J., Balasuriya U.B. (2006). Equine viral arteritis. Adv. Exp. Med. Biol..

[2] Nitschke M., Korte T., Tielesch C., Ter-Avetisyan G., Tunnemann G., Cardoso M.C., Veit M., Herrmann A. (2008). Equine arteritis virus is delivered to an acidic compartment of host cells via clathrin-dependent endocytosis. Virology.

[3] Snijder E.J., Meulenberg J.J. (1998). The molecular biology of arteriviruses. J. Gen. Virol..

[4] Snijder E.J., Dobbe J.C., Spaan W.J. (2003). Heterodimerization of the two major envelope proteins is essential for arterivirus infectivity. J. Virol..

[5] Snijder E.J., van Tol H., Pedersen K.W., Raamsman M.J., de Vries A.A. (1999). Identification of a novel structural protein of arteriviruses. J. Virol..

[6] Wieringa R., De Vries A.A., Post S.M., Rottier P.J. (2003). Intra- and intermolecular disulfide bonds of the GP2b glycoprotein of equine arteritis virus: Relevance for virus assembly and infectivity. J. Virol..

[7] Wieringa R., de Vries A.A., van der Meulen J., Godeke G.J., Onderwater J.J., van Tol H., Koerten H.K., Mommaas A.M., Snijder E.J., Rottier P.J. (2004). Structural protein requirements in equine arteritis virus assembly. J. Virol..

[8] Verheije M.H., Welting T.J., Jansen H.T., Rottier P.J., Meulenberg J.J. (2002). Chimeric arteriviruses generated by swapping of the M protein ectodomain rule out a role of this domain in viral targeting. Virology.

[9] Dobbe J.C., van der Meer Y., Spaan W.J., Snijder E.J. (2001). Construction of chimeric arteriviruses reveals that the ectodomain of the major glycoprotein is not the main determinant of equine arteritis virus tropism in cell culture. Virology.

[10] Thaa B., Kabatek A., Zevenhoven-Dobbe J.C., Snijder E.J., Herrmann A., Veit M. (2009). Myristoylation of the arterivirus E protein: the fatty acid modification is not essential for membrane association but contributes significantly to virus infectivity. J. Gen. Virol..

[11] de Vries A.A., Raamsman M.J., van Dijk H.A., Horzinek M.C., Rottier P.J. (1995). The small envelope glycoprotein (GS) of equine arteritis virus folds into three distinct monomers and a disulfide-linked dimer. J. Virol..

[12] Wieringa R., de Vries A.A., Rottier P.J. (2003). Formation of disulfide-linked complexes between the three minor envelope glycoproteins (GP2b, GP3, and GP4) of equine arteritis virus. J. Virol..

[13] Ellgaard L., Helenius A. (2001). ER quality control: Towards an understanding at the molecular level. Curr. Opin. Cell Biol..

[14] Doms R.W., Lamb R.A., Rose J.K., Helenius A. (1993). Folding and assembly of viral membrane proteins. Virology.

[15] Braakman I., van Anken E. (2000). Folding of viral envelope glycoproteins in the endoplasmic reticulum. Traffic.

[16] Veit M., Kabatek A., Tielesch C., Hermann A. (2008). Characterization of equine arteritis virus particles and demonstration of their hemolytic activity. Arch. Virol..

[17] Kyte J., Doolittle R.F. (1982). A simple method for displaying the hydropathic character of a protein. J. Mol. Biol..

[18] Skehel J.J., Cross K., Steinhauer D., Wiley D.C. (2001). Influenza fusion peptides. Biochem. Soc. Trans..

[19] Tamm L.K., Han X., Li Y., Lai A.L. (2002). Structure and function of membrane fusion peptides. Biopolymers.

[20] Baneyx F., Mujacic M. (2004). Recombinant protein folding and misfolding in *Escherichia coli*. Nat. Biotechnol..

[21] Ventura S., Villaverde A. (2006). Protein quality in bacterial inclusion bodies. Trends Biotechnol..

[22] Singh S.M., Panda A.K. (2005). Solubilization and refolding of bacterial inclusion body proteins. J. Biosci. Bioeng..

